# Supervised Toothbrushing and Silver Diamine Fluoride Application of Rohingya Children in a Refugee Camp in Bangladesh

**DOI:** 10.1016/j.identj.2025.100929

**Published:** 2025-08-05

**Authors:** Khaleda Zaheer, Tahmina Zerin, James Coughlan, Shuvashis Saha, Elsa Delgado-Angulo, Elham Kateeb

**Affiliations:** a Refugee Crisis Foundation, London, UK; b Refugee Crisis Foundation, Cox’s Bazar, Bangladesh; c Health Organisation, Policy and Economics Group, University of Manchester, Manchester, UK; d Dental Public Health Group, Centre for Host Microbiome Interactions, Faculty of Dentistry, Oral & Craniofacial Sciences, King’s College London, London, UK; e Departamento Académico de Odontología Social, Facultad de Estomatología, Universidad Peruana Cayetano Heredia, Lima, Peru; f Oral Health Research and Promotion Unit, Al-Quds University, Jerusalem, Palestine; g FDI World Dental Federation, Public Health Committee, Geneva, Switzerland

**Keywords:** Oral health, Refugees, Refugee camps, Dental health surveys

## Abstract

**Introduction and aims:**

Previous studies have highlighted the high burden of oral diseases in refugee camps, but there are few robustly evaluated school-based oral health programmes in these settings. The objective of this study was to pilot a school-based toothbrushing, handwashing, and silver diamine fluoride (SDF) application in a refugee camp in Bangladesh. It hosts the world’s largest number of refugees with over 1 million Rohingya from Myanmar.

**Methods:**

A prospective cohort study that consisted of a multipronged oral health intervention: supervised daily toothbrushing, handwashing, and SDF application of active caries in the primary and permanent dentition. Clinical examination of the children and child and parental questionnaires were completed at baseline and the 6-month follow-up.

**Results:**

A total of 176 children were assessed at baseline, with a follow-up rate of 87.5%. The number of children reporting regular toothbrushing and handwashing significantly increased between baseline and 6-month follow-up, with a corresponding fall in plaque and bleeding scores. There was a 54.3% caries arrest rate in teeth where SDF was applied.

**Conclusion:**

Supervised toothbrushing and SDF application in learning centres in refugee camps can improve the oral health of refugee children and instil healthy hygiene habits. Further research is warranted to robustly assess the long-term impact of this intervention and scalability across humanitarian settings.

**Clinical Relevance:**

In the absence of accessible oral health care in refugee camps, low-resource oral health interventions can safeguard oral health and instil healthy hygiene habits from a young age.

## Introduction

Over 122.6 million people are forcibly displaced due to persecution, violence, human rights violations, conflict, or events seriously disturbing public order.^1^ Low- and middle-income countries host 75% of the world’s refugees, of which there are 43.4 million, and this has tripled in the last decade. The United Nations High Commissioner for Refugees predicts that the number of forcibly displaced people including refugees is likely to increase due to new and ongoing conflicts across the globe.^2^

Bangladesh hosts the world’s largest refugee settlement with over 1 million Rohingya refugees from neighbouring Myanmar.^3^ This is one of the largest protracted refugee crises in the world. The Rohingya have been repeatedly targeted and persecuted over the last five decades, resulting in large-scale forced displacement. Following a massive wave of violence in the Rakhine state in Myanmar, the largest displacement took place in August 2017, forcing 742,000 Rohingya who took refuge in Bangladesh. The Rohingya in Bangladesh reside in 33 congested camps in the densely populated and climate-vulnerable district of Cox’s Bazar, as well as on the island of Bhasan Char. The Rohingya refugees are reliant on lifesaving humanitarian assistance, and the Government of Bangladesh is working towards repatriation to Myanmar.^4^

Children make up 52% of the over 1 million Rohingya, and they are disproportionately affected by the crisis in Myanmar and remain vulnerable.^5^ Approximately 5,500 learning centres have been set up in the camps to provide education to the children using the Myanmar curriculum in the Myanmar language. It has been recognised that priority should be given to community-based health services on disease prevention and health promotion.^4^

Oral disease disproportionately affects marginalised and vulnerable populations, which includes refugees. A recent study investigated current policies, and activities related to refugee oral health (OH) reported that OH is a low priority.^6^ Refugees are entitled to oral healthcare, and this is a basic refugee health right, and therefore, stakeholders are being urged to integrate OH into overall health and well-being for refugees.^7^

The social determinants of OH include access to safe water, sanitation, and hygiene, which can be challenging in humanitarian settings. Midstream initiatives that create supportive conditions, such as supervised toothbrushing programmes, can reduce the burden of most oral diseases that are preventable.^8^ Clean water scarcity, limited access to sanitation, and lack of hygiene, a challenge in refugee camps, contribute to several childhood diseases which impact on academic performance.^9^^,^^10^

Adopting healthy hygiene habits at an early age is a crucial step that contributes to healthy development and reduction of preventable diseases. The World Health Organization developed the School Health Initiative 25 years ago,^11^ which Kwan et al^12^ adapted by integrating OH promotion into this programme. A review of OH promotion initiatives in schools in developing countries reported that multicomponent strategies, toothbrushing and oral hygiene instructions, had promising results by significantly reducing plaque index and caries.^13^

Several OH preventative models have been tested to improve oral hygiene practices. The Fit for School programme including daily toothbrushing, handwashing, and deworming demonstrated success in preventing caries; however, it did not address existing cavitated carious lesions.^14^ Turton et al^15^ proposed and tested the Healthy Kids Cambodia (HKC), which aims to prevent the initiation and progression of caries with a graduated delivery of care from simplex to complex dental treatment. This facilitates allocation of limited resources. Children in the schools are triaged using a three-tiered system: level one includes daily toothbrushing and handwashing and application of silver diamine fluoride (SDF) to arrest caries in primary teeth; level two includes the atraumatic restorative technique; and level three is comprehensive oral rehabilitation in a dental clinic.^15^^,^^16^ All three levels of the HKC require substantial resources including the dental workforce which is a barrier to upscaling, and therefore, Turton et al^17^ tested level one and reported 87.3% caries’ arrest rate.^17^

In this study, we adopted and piloted level one of the HKC model in the refugee camps in Cox’s Bazar. The fundamental requirement to implement such a programme required access to clean water to enable hygiene activities, ie, handwashing and toothbrushing. Although handwashing is taught in the camps, there is limited access to clean water in the learning centres for the children. Furthermore, as the Rohingya refugees are stateless, they have had limited or no access and exposure to dental care, and this is further exacerbated by a similar situation in the refugee camps in Bangladesh.^18^ Therefore, due to extremely limited resources and funding in humanitarian settings, in this study, the intervention included daily toothbrushing with fluoridated toothpaste, daily handwashing, healthy eating, and SDF application to cavitated carious lesions in the primary and permanent dentition.

The objective of this study was to assess the feasibility and effect of a pilot supervised toothbrushing, handwashing, and SDF intervention on oral hygiene and handwashing practice and OH status of children in a learning centre in the refugee camp in Cox’s Bazar, Bangladesh.

## Material and methods

### Study design

This study was a pilot prospective cohort study, which aimed to explore the feasibility and impact on oral hygiene and handwashing practice, and OH status, of a supervised toothbrushing, handwashing, and SDF intervention for Rohingya children at a learning centre in the refugee camp in Cox’s Bazar, Bangladesh. Ethical approval was obtained from North South University, Dhaka, Bangladesh (Ref: #2024/OR-NSU/IRB/0409).

### Setting and participants

There are a total of 33 camps in Bangladesh hosting Rohingya refugees. This was a pilot study conducted in a learning centre in camp 13, equivalent to a school. The site was selected based on an established partnership between the local non-governmental organisation managing the learning centre and the UK-based charity, Refugee Crisis Foundation, which enabled logistical support, local authority approval, and access. The children enrolled in the learning centre are aged 3-14 years.

Our study population comprises all children attending a single learning centre. The parents or guardians were invited to participate in the study with information provided about the study, and verbal consent was obtained. As a pilot study, we did not seek to determine a clinically or statistically significant effect. Therefore, we did not carry out a power calculation to determine a minimum sample size, and all eligible children were invited to participate. The learning centre was chosen due to existing links through the charity, Refugee Crisis Foundation, with the local learning centre allowing installation of washing facilities and implementation of the intervention.

### Data collection

Data collection comprises two stages: firstly, through structured interviews with the children and the parents or guardians, followed by clinical examination of the children.

The questionnaire was based on existing OH questionnaires used in marginalised and underserved settings (Supplemental Information).^19^^,^^20^ Data were collected on demographic, body mass index (BMI),^21^ OH behaviours, parental attitudes and knowledge, and access to oral healthcare. Additional questions on handwashing were adapted from the Global School-based Student Health Survey.^22^ Parental knowledge, attitudes, and behaviours were assessed using single-item questions developed for low-literacy refugee populations.

As the Rohingya language is understood in oral form in the camps, the questionnaire was verbally translated from English into Rohingya by trained Rohingya enumerators during structured interviews. These enumerators were themselves refugees residing in the camps and were fluent in Rohingya, Bangla, and English. They had prior experience conducting surveys and received dedicated training for this study.

The clinical examination and SDF application were conducted by 2 examiners, authors TZ and SS, who are local Bangladeshi dentists providing oral healthcare in the camps for the UK-registered charity, Refugee Crisis Foundation. Examinations took place in the learning centre, with children seated for examination, and visibility enhanced by use of an artificial headlight and by drying teeth with cotton-wool rolls. OH screening was based on the World Health Organization’s Basic Methods for Dental Surveys for recording decayed, missing, and filled teeth (dmft/DMFT), and pulp involvement, ulceration, fistula, and abscess (pufa/PUFA) indices.^23^ The number of primary and permanent teeth with active carious lesions (defined as cavitated lesions not involving the pulp), arrested carious lesions (hard or black lesions), pulpal involvement (meets any of the four PUFA criteria), and active infection (fistula or abscess of the PUFA index) were recorded, as well as the number of missing or extracted and filled teeth. Periodontal outcomes included modified plaque and bleeding scores using the index teeth 16, 11, 26, 36, 41, and 46.

The two examiners underwent 2 hours of remote training prior to data collection and were calibrated. Inter-examiner (intraclass correlation coefficient = 0.894) and intra-examiner agreements (intraclass correlation coefficient = 0.922) were measured by intraclass correlation with a 2-way mixed-effects model.

### Intervention

The intervention consisted of three stages. Firstly, washing facilities were constructed which included dedicated stations for handwashing and toothbrushing in the learning centre. There were separate training sessions with the parents and children, and the teachers. This included handwashing, toothbrushing, healthy eating, and tobacco cessation, as its use is highly prevalent in the Rohingya refugees,^18^ and the local dental team had reported its use by children. The teachers were provided with aids, including models and posters, to facilitate demonstration of toothbrushing to the children. The toothbrushes were labelled with the child’s name and stored in the classroom. The teachers received the training twice during the study period.

Secondly, daily toothbrushing with fluoridated toothpaste and handwashing took place at the washing stations with the supervision of the trained teachers. This was scheduled formally as a daily activity called ‘Healthy Smiles’ into the classroom’s schedule. Throughout the 6-month intervention period, teachers provided daily reminders and supervision on correct toothbrushing technique, handwashing, and healthy eating. These activities were integrated into the school routine to ensure continuity, encourage compliance, and reinforce behaviour change among children.

The third stage was the OH screening of the children by the trained and calibrated dentists, and parent and child questionnaire by Rohingya enumerator, at baseline and the 6-month follow-up. At baseline, SDF (38%, Topamine) was applied to primary and permanent teeth with active carious lesions without pulpal involvement, in accordance with previously published protocols.^17^

### Statistical analysis

All statistical analyses were conducted on STATA 18. Descriptive statistics were calculated from counts and proportions, while the differences in clinical outcomes (bleeding index, plaque index, and teeth with active caries) were compared with mean differences, with statistical significance determined by the Wilcoxon signed-rank test for continuous or ordered outcomes, and the χ^2^ test for categorical outcomes. In this pilot, clinical outcomes were recorded at the child level as opposed to tooth level, precluding tooth-level follow-up. Initial subgroup analysis by sex, parental education level, brushing frequency, brushing instrument, dentifrice, and person responsible for brushing the child’s teeth can be found in Supplemental Material.

## Results

One hundred and seventy-six children and parents completed the questionnaire and were examined at baseline. They were slightly more females than males, in early years of education, and came from families where most parents stayed at home and had informal or no education; it was also observed that almost half of the sample were underweight (Table 1). Of them, 154 (87.5%) attended the follow-up visit, and 12.5% were lost to follow-up.

**Table 1:** Sociodemographic characteristics at baseline (*n* = 176).

	*n*	%
Gender		
Male	84	47.7
Female	92	52.3
Age (mean/SD)	8.8	2.6
Education		
Kindergarten	52	29.5
Grade 1	30	17.1
Grade 2	51	29.0
Grade 3	35	19.9
Grade 4	8	4.5
BMI		
Underweight	79	44.9
Normal	77	43.7
Overweight	20	11.4
Parent’s employment status		
Nil	29	16.5
Stay at home spouse	116	65.9
Labourer/Skilled/Unskilled work	22	12.5
Professional/Managerial	9	5.1
Parent’s education level		
None/Informal	145	82.4
School	24	13.6
College and above	7	4.0
Parent had access to dental care in Myanmar	12	6.8

BMI, body mass index.

After the 6 months of intervention, there were favourably significant changes in the children’s toothbrushing frequency and the aids chosen for cleaning their teeth as well as handwashing before eating; and among their parents, there were statistically significant differences in their perception of their child’s OH, having received information on their child’s OH, knowledge on where to access dental care, who cleans the child’s teeth, and their own choice of aid for cleaning their teeth. In all, there was an increase in the frequency of children brushing their teeth twice a day and using a toothbrush for cleaning their teeth; their sugary beverages’ consumption shows some changes, although those are difficult to characterise. In addition, a greater proportion of children adopted the habit of washing their hands before meals by reporting ‘always’ or ‘most of the time’ (Table 2).

**Table 2:** Baseline and follow-up behaviours (*n* = 154).

	Baseline		Follow-up		*P* value*
	*n*	%	*n*	%	
Toothbrushing frequency					.002
Twice a day	96	62.7	115	75.2	
Once a day	43	28.1	30	19.6	
Few times a week	13	8.5	8	5.2	
Never	1	0.7	0	0.0	
Aids for cleaning teeth					.038
Brush	110	71.4	133	86.4	
Other (miswak or finger)	44	28.6	21	13.6	
Type of dentifrice					.223
Toothpaste	103	66.5	99	63.9	
Toothpaste combined with other	9	5.8	29	18.7	
Other (salt, charcoal, and sand)	43	27.7	27	17.4	
Frequency of sugar-sweetened drink in the past week					.028
None	48	36.9	39	30.0	
1-3 times	63	48.4	69	53.1	
4-6 times	17	13.1	15	11.5	
One per day	1	0.8	6	4.6	
4 or more per day	1	0.8	1	0.8	
Frequency of handwashing before eating					.027
Always	144	92.9	134	86.5	
Most of the time	6	3.9	20	12.9	
Sometimes	3	1.9	0	0.0	
Rarely	2	1.3	1	0.6	
Frequency of handwashing after using the toilet					.757
Always	58	37.7	118	76.6	
Most of the time	30	19.5	32	20.8	
Sometimes	29	18.8	4	2.6	
Rarely	35	22.7	0	0.0	
Never	2	1.3	0	0.0	
Ever tried *paan*					.004
Yes	19	12.3	6	3.9	
Parent’s confidence on their knowledge of child’s OH					.424
Very confident	150	95.5	92	58.6	
Somewhat confident	7	4.5	23	14.7	
Not very confident	0	0.0	25	15.9	
Not confident at all	0	0.0	17	10.8	
Parent’s perception of child’s OH status					.001
Excellent	88	56.4	25	16.0	
Good	36	23.1	115	73.7	
Fair	18	11.5	10	6.4	
Poor	14	9.0	6	3.9	
Has parent received information about child’s OH in Bangladesh					.031
Yes	44	28.0	61	38.9	
Does parent know where to access dental care in Bangladesh					.026
Yes	30	19.1	76	48.4	
Has child received dental care since arriving in Bangladesh					.521
Yes	5	3.4	11	7.4	
Who cleans child’s teeth					.035
Child	140	89.8	131	77.6	
Adult	8	5.1	25	16.0	
Adult and child together	7	4.5	6	3.8	
No one	1	0.6	4	2.6	
Parent’s aid for cleaning teeth					.029
Brush	134	85.9	131	84.0	
Other (finger or miswak)	22	14.1	25	16.0	
Parent’s type of dentifrice					.206
Toothpaste	87	56.9	114	74.5	
Toothpaste combined with others	49	32.0	12	7.8	
Others (salt, charcoal, or sand)	17	11.1	27	17.7	

OH, oral health.

⁎ Chi-square test.

There was also a shift in the parent’s perception of their child’s OH, with a greater proportion rating it as ‘good’ or ‘excellent’ in comparison to the baseline questionnaire. In addition, a greater proportion of parents reported having received information about their child’s OH and knowing where to access dental care as compared to their initial responses. Furthermore, there was a significant shift in oral hygiene practices, with a greater proportion of parents being involved in child’s oral hygiene routine (Table 2).

**Fig. 1:**
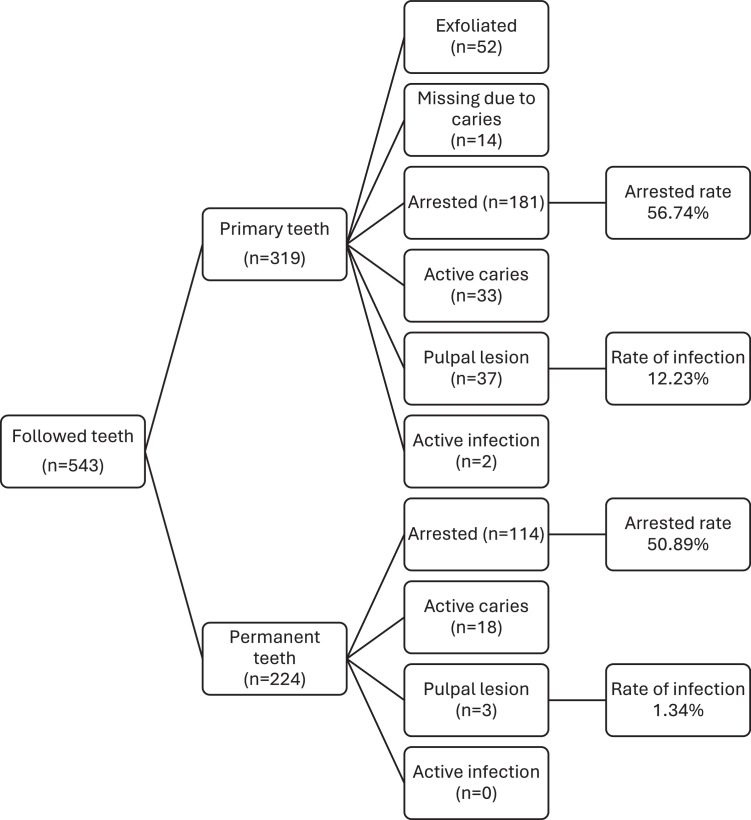
Tooth-level outcomes by dentition (*n* = 84).

Table 3 shows the differences in clinical outcomes after the intervention. In summary, there was a significant reduction in the plaque index, bleeding score, and number of teeth with active carious lesions, which derived from an increase in the number of arrested carious lesions after SDF application. On average, the overall arrested rate was 54.3%: 56.7% in primary teeth and 50.9% in permanent teeth (Fig. 1).

**Table 3:** Comparison of clinical indicators at baseline and follow-up (*n* = 151).

	Baseline		Follow-up		*P* value*
	Mean	SD	Mean	SD	
Plaque index	3.27	1.17	2.44	1.42	<.001
Bleeding score	0.76	0.32	0.12	0.25	<.001
Number of primary teeth with cavitated lesions	2.11	2.06	0.26	0.80	<.001
Number of primary teeth with arrested lesions	0.08	0.45	1.25	1.71	<.001
Number of pulpally involved primary teeth	0.30	0.88	0.29	0.78	.918
Number of primary teeth with active infection	0.03	0.27	0.02	0.14	.750
Number of permanent teeth with cavitated lesions	1.47	1.72	0.18	0.54	<.001
Number of permanent teeth with arrested lesions	0.02	0.18	0.91	1.21	<.001
Number of pulpally involved permanent teeth	0.01	0.08	0.02	0.18	1.000
Number of permanent teeth with active infection	0.00	0.00	0.03	0.34	.250

⁎ Wilcoxon-signed.

## Discussion

To the best of our knowledge, this is the first pilot programme implementing a supervised toothbrushing, handwashing, and SDF application for children in refugee camps. We find promising early results; the intervention was widely accepted by children and parents, with a high 6-month follow-up and good engagement with the data collection. Participants reported significant improvements in brushing habits and handwashing practices between baseline and 6-month follow-up, and while more time is needed to fully assess the long-term clinical outcomes such as caries’ recurrence, proxy short-term clinical outcomes such as plaque and bleeding scores reduced significantly following the intervention. This suggests that toothbrushing may successfully be integrated with other water, sanitation, and hygiene interventions,^24^ and a wider clinical trial is warranted to assess the efficacy of this low-cost intervention in other camps and over a longer follow-up period.

Overall, the findings from this pilot study are consistent with previous literature on the prevalence of caries in refugee camps. A study assessing untreated caries in Libyan children living in conflict zones and camps reported the mean number of carious teeth as 1.20 and 0.68 in primary and permanent teeth, respectively.^25^ This caries’ prevalence was significantly higher in the Rohingya children in this study as the mean number of cavitated lesions in primary and permanent was 2.11 and 1.47, respectively.

The clinical element of the intervention, involving SDF application, was also successful, with 54.3% arrest rate. An observational cohort study among school children in Cambodia evaluating SDF application at baseline with toothbrushing and compared to 9 months after toothbrushing found that due to early application of SDF, 9 out of 10 teeth had caries arrested and no pulpal involvement. Timely application of SDF reduces in the incidence of pulpally involved lesions.^17^ The application of SDF in humanitarian settings has multiple advantages, including ease of application, low cost, efficacy, and reduced infections from active caries. Access to dental care is limited in refugee camps,^18^ and, as reported, most of the refugees have never seen a dentist, and therefore, traditional dental procedures are not only expensive and require access to a dentist, but can be traumatising for children with no experience of dental care.

Biannual application of SDF has been recommended as the most effective regime, and this will be offered to the children recruited in this study. It has also been highlighted in other studies that optimal plaque control is important in the control of caries’ progression following SDF as food trapping can affect the remineralisation action of saliva.^26^ Therefore, continued supervised toothbrushing is essential to maintain and further improve the OH of the refugee children. At follow-up, parents or caregivers reported greater involvement in their child’s oral hygiene routine. This is a positive finding, as current guidelines from ‘Delivering Better Oral Health’ recommend that it is good practice for a parent or carer to assist and supervise toothbrushing for children aged 7 and above who are at higher risk of dental caries.^27^

In addition to OH measures, BMI was recorded to support a comprehensive assessment of the child’s general health status. This inclusion was based on the understanding that poor OH can affect dietary intake and nutritional status,^28^ particularly in prolonged refugee settings. While BMI was not analysed as an outcome of the intervention, it was collected to contextualise the overall health of participants and inform future programme design. In this study, 45% of the children were underweight. Children in conflict zones are vulnerable to malnourishment^29^ due to key sociodemographic factors, and inadequate nutrition, sanitation, and hygiene are crucial factors.

In this study, there was positive engagement from parents, teachers, and children with the intervention and data collection. However, there were several challenges with programme implementation due to the monsoon season and cyclones, and this resulted in significant delays in the construction of the washing stations and scheduling administration of the surveys and training sessions. Generally, health-promoting initiatives in schools require regular funding and resources to sustain it, and this can be challenging in refugee camps, especially in the current climate with significant cuts in aid.

This study has several limitations. Firstly, it relies on before–after comparisons to evaluate the impact on the short-term outcomes due to the lack of a control group. There was initially planned to be a comparison school; however, natural disasters resulted in significant delays in the construction of the washing stations and scheduling administration of the surveys and training sessions, highlighting the challenges of conducting research in humanitarian settings. In addition, we do not have variation in the timing or exposure to each element of the intervention, so we cannot ascertain in this pilot study whether the treatment, brushing, or provision of oral hygiene products is driving the changes in outcomes. However, as a pilot study, these are secondary issues.

Another limitation of the study is that parental knowledge, attitudes, and behaviours were assessed using single-item questions, which may not fully capture the multidimensional nature of these constructs. However, the items were developed to ensure survey feasibility in a low-literacy refugee setting, and the results from this study can serve as a basis for future research exploring parental OH-related behaviour and knowledge.

These results have implications for future research. The intervention was successfully delivered, showed some early signs of effectiveness, and was well accepted by children and parents. As such, a full trial is warranted to evaluate the effectiveness of the intervention across a wider population in the camp and with a longer follow-up.

## Conclusions

The protracted situation of the Rohingya in Bangladesh and lack of access to OH care highlight the need for community-based OH promotion and prevention programmes to safeguard OH. We show that an integrated community-based OH intervention can improve short-term clinical outcomes and warrant further trials to assess its long-term effects on OH.

## Author contributions

Khaleda Zaheer, James Coughlan, and Elham Kateeb contributed to the conception and design of the study, interpretation of data, drafting the article, and final approval of the version to be submitted, and James Coughlan also contributed to the data analysis. Tahmina Zerin and Shuvashis Saha contributed to the acquisition of data, drafting the article, and final approval of the version to be submitted. Elsa Delgado-Angulo contributed to data analysis, drafting the article, and final approval of the version to be submitted.

## Funding

This research was partially funded by the FDI Refugee Oral Health Project. James Coughlan receives funding from the NIHR Doctoral Training Programme for Primary Care Clinicians. The views expressed are those of the author(s) and not necessarily those of the NIHR or the Department of Health and Social Care.

## Conflict of interest

The authors declare that they have no known competing financial interests or personal relationships that could have appeared to influence the work reported in this article.

## Appendix Supplementary materials

Image, application 1

## Footnotes

Supplementary material associated with this article can be found in the online version at doi:10.1016/j.identj.2025.100929.
