# Supplementary material for: Supervised Toothbrushing and Silver Diamine Fluoride Application of Rohingya Children in a Refugee Camp in Bangladesh
Source: Int Dent J. 2025 Aug 5;75(5):100929. doi: 10.1016/j.identj.2025.100929 (PMC12345283; doi:10.1016/j.identj.2025.100929)
Supplement: Supplementary file 1 [file mmc1.docx]

**Supplemental File**

**Child and parental questionnaire**

| **Child questionnaire** |
| --- |
| 1. Child Height (kg) |
| 1. Child Weight (cm) |
| 1. Gender |
| Male |
| Female |
| 1. Class |
| Kindergarten |
| Grade 1 |
| Grade 2 |
| Grade 3 |
| Grade 4 |
| 1. How often do you clean your teeth at home? |
| Twice a day |
| Once a day |
| A few times a week |
| Once a week |
| Never |
| 1. Do you have a toothbrush? |
| Yes |
| No |
| 1. What do you use to clean your teeth with? |
| Brush |
| Finger |
| Miswak |
| Cloth |
| Other |
| 1. What type of dentifrice do you use? |
| Toothpaste |
| Salt |
| Charcoal |
| Sand |
| Water only |
| Other |
| 1. During the past 7 days, how many times did you drink a can, bottle, or glass of a sugar-sweetened drink? |
| I did not drink sugar-sweetened drinks during the past 7 days |
| 1 to 3 times during the past 7 days |
| 4 to 6 times during the past 7 days |
| 1 time per day |
| 2 times per day |
| 3 times per day |
| 4 or more times per day |
| 1. Have you ever tried paan? |
| Yes |
| No |
| 1. During the past 30 days, how often did you wash your hands before eating? |
| Never |
| Rarely |
| Sometimes |
| Most of the time |
| Always |
| 1. During the past 30 days, how often did you wash your hands after using the toilet or latrine? |
| Never |
| Rarely |
| Sometimes |
| Most of the time |
| Always |
| **Parental questionnaire** |
| 1. What’s your employment status? |
| Nil |
| Stay-at-home spouse |
| Labourer / skilled/ unskilled work |
| Professional/ managerial |
| 1. What’s your highest education level? |
| None |
| Less than high school |
| High school |
| High school with some college |
| College graduate and above |
| 1. How confident do you feel in your knowledge of your child's dental health? |
| Very confident |
| Somewhat confident |
| Not very confident |
| Not confident at all |
| 1. How would you rate your child's oral health? |
| Excellent |
| Good |
| Fair |
| Poor |
| 1. Have you received information on your child's oral health since arriving in Bangladesh? |
| Yes |
| No |
| 1. Do you know where to access dental care for your child in Bangladesh? |
| Yes |
| No |
| 1. Did you have access to dental care in Myanmar? |
| Yes |
| No |
| 1. Who usually cleans your child’s teeth? |
| Your child |
| An adult |
| An adult and child together |
| No one |
| 1. Has your child received dental care or treatment since arriving in Bangladesh? |
| Yes |
| No |
| 1. What do you use to clean your teeth with? |
| Brush |
| Finger |
| Miswak |
| Cloth |
| Other |
| 1. What type of dentifrice do you use? |
| Toothpaste |
| Salt |
| Charcoal |
| Sand |
| Water only |
| Others |

**Supplemental File**

Table S1. Change in Plaque Index according to sociodemographic characteristics and OH-related behaviours

|  |  | Plaque Index Baseline | | | Plaque Index Follow-up | | | Difference in Plaque Index | | |
| --- | --- | --- | --- | --- | --- | --- | --- | --- | --- | --- |
|  |  | Median | (IQ range) | p-value* | Median | (95%CI) | p-value* | Median | (95%CI) | p-value* |
| Overall | | 3.5 | (2.7;4.0) |  | 2.5 | (1.2;3.5) |  | -0.8 | (-2.0;0.3) | <0.001 |
| Sex | |  |  | 0.594 |  |  | 0.572 |  |  | 0.914 |
|  | Male | 3.5 | (2.8;4.0) |  | 2.8 | (1.2;3.7) |  | -0.7 | (-2.2;0.5) |  |
|  | Female | 3.4 | (1.5;4.0) |  | 2.5 | (1.5;3.2) |  | -1.1 | (-1.7;0.3) |  |
| Parental Education level | | |  | 0.087 |  |  | 0.074 |  |  | 0.058 |
|  | None/Informal | 3.5 | (2.8;4.0) |  | 2.5 | (1.2;3.6) |  | -0.8 | (-1.9;0.3) |  |
|  | School | 2.7 | (1.3;4.1) |  | 2.8 | (2.2;3.5) |  | -0.2 | (-1.2;0.7) |  |
|  | College and above | 4.0 | (3.3;4.5) |  | 1.8 | (0.7;3.3) |  | -2.2 | (-2.7;-0.8) |  |
| Frequency of toothbrushing | | |  | 0.217 |  |  | 0.930 |  |  | 0.631 |
|  | Few times a week | 3.3 | (2.5;4.0) |  | 2.5 | (0.8;3.3) |  | -0.8 | (-2.3;0.3) |  |
|  | Once daily | 3.7 | (2.7;4.3) |  | 2.8 | (2.0;4.0) |  | -0.8 | (-1.7;0.5) |  |
|  | Twice daily | 3.7 | (3.2;4.5) |  | 2.8 | (2.2;3.7) |  | -0.8 | (-1.2;0.3) |  |
| Instrument used to clean teeth | | |  | 0.879 |  |  | 0.677 |  |  | 0.881 |
|  | Toothbrush | 3.5 | (2.8;4.0) |  | 2.5 | (1.2;3.3) |  | -1.0 | (-2.0;0.3) |  |
|  | Other | 3.0 | (2.2;3.8) |  | 2.7 | (2.0;3.7) |  | 0.0 | (-1.0;0.8) |  |
| Choice of Dentifrice | |  |  | 0.494 |  |  | 0.262 |  |  | 0.277 |
|  | Toothpaste | 3.5 | (2.7;4.0) |  | 2.5 | (0.8;3.7) |  | -0.8 | (-2.0;0.3) |  |
|  | Toothpaste + others | 3.1 | (1.0;4.7) |  | 2.8 | (2.2;3.2) |  | -0.7 | (-2.3;0.7) |  |
|  | Other | 3.3 | (2.7;4.0) |  | 2.7 | (2.0;3.3) |  | -0.8 | (-1.7;0.0) |  |
| Who cleans child teeth | |  |  | 0.321 |  |  | 0.337 |  |  | 0.442 |
|  | Child | 3.5 | (2.7;4.0) |  | 2.5 | (1.5;3.6) |  | -0.8 | (-1.7;0.3) |  |
|  | Adult | 3.3 | (2.3;3.8) |  | 1.5 | (0.4;2.5) |  | -1.3 | (-2.4;-0.2) |  |
|  | Child and adult together | 3.8 | (3.7;4.0) |  | 3.0 | (1.2;4.2) |  | 0.0 | (-2.8;0.3) |  |

* Wilcoxon-signed

Table S2. Change in Bleeding Score according to sociodemographic characteristics and OH-related behaviours

|  |  | Bleeding Score Baseline | | | | | Bleeding Score Follow-up | | | Difference in Bleeding Score | | |
| --- | --- | --- | --- | --- | --- | --- | --- | --- | --- | --- | --- | --- |
|  |  | Median | (IQ range) | | p-value* | | Median | (95%CI) | p-value* | Median | (95%CI) | p-value* |
| Overall | | 0.9 | (0.7;1.0) | |  | | 0.0 | (0.0;0.2) |  | -0.8 | (-1.0;-0.5) | <0.001 |
| Sex | |  |  | | 0.216 | |  |  | 0.190 |  |  | 0.850 |
|  | Male | 1.0 | (0.7;1.0) | |  | | 0.0 | (0.0;0.2) |  | -0.7 | (-1.0;-0.5) |  |
|  | Female | 0.8 | (0.7;1.0) | |  | | 0.0 | (0.0;0.0) |  | -0.8 | (-1.0;-0.3) |  |
| Parental Education level | | | |  | | 0.054 |  |  | 0.597 |  |  | 0.481 |
|  | None/Informal | 1.0 | (0.7;1.0) | |  | | 0.0 | (0.0;0.2) |  | -0.8 | (-1.0;-0.5) |  |
|  | School | 0.8 | (0.6;1.0) | |  | | 0.0 | (0.0;0.2) |  | -0.7 | (-0.8;-0.2) |  |
|  | College and above | 1.0 | (1.0;1.0) | |  | | 0.0 | (0.0;0.0) |  | -1.0 | (-1.0;-0.8) |  |
| Frequency of toothbrushing | | | |  | | 0.876 |  |  | 0.414 |  |  | 0.535 |
|  | Few times a week | 1.0 | (0.7;1.0) | |  | | 0.0 | (0.0;0.0) |  | -0.8 | (-1.0;-0.5) |  |
|  | Once daily | 0.8 | (0.7;1.0) | |  | | 0.0 | (0.0;0.3) |  | -0.7 | (-1.0;-0.3) |  |
|  | Twice daily | 1.0 | (0.7;1.0) | |  | | 0.0 | (0.00;0.2) |  | -0.7 | (-1.0;-0.5) |  |
| Instrument used to clean teeth | | | |  | | 0.767 |  |  | 0.350 |  |  | 0.640 |
|  | Toothbrush | 1.0 | (0.7;1.0) | |  | | 0.0 | (0.0;0.2) |  | -0.7 | (-1.0;-0.5) |  |
|  | Other | 0.8 | (0.7;1.0) | |  | | 0.0 | (0.0;0.0) |  | -0.8 | (-0.8;-0.3) |  |
| Choice of Dentifrice | |  |  | | 0.536 | |  |  | 0.752 |  |  | 0.778 |
|  | Toothpaste | 0.8 | (0.7;1.0) | |  | | 0.0 | (0.0;0.2) |  | -0.7 | (-1.0;-0.5) |  |
|  | Toothpaste + others | 1.0 | (0.0;1.0) | |  | | 0.2 | (0.0;0.2) |  | -0.8 | (-1.0;0.0) |  |
|  | Other | 0.8 | (0.7;1.0) | |  | | 0.0 | (0.0;0.0) |  | -0.8 | (-1.0;-0.5) |  |
| Who cleans child teeth | |  |  | | 0.716 | |  |  | 0.862 |  |  | 0.141 |
|  | Child | 1.0 | (0.7;1.0) | |  | | 0.0 | (0.0;0.2) |  | -0.8 | (-1.0;-0.5) |  |
|  | Adult | 0.6 | (0.5;1.0) | |  | | 0.0 | (0.0;0.0) |  | -0.7 | (-0.9;-0.3) |  |
|  | Child and adult together | 1.0 | (0.5;1.0) | |  | | 0.0 | (0.0;0.0) |  | -0.7 | (-1.0;0.0) |  |

* Wilcoxon-signed
